# Role of Prophylactic Dexamethasone Before Thyroidectomy in Reducing Postoperative Pain, Nausea and Vomiting

**DOI:** 10.7759/cureus.4735

**Published:** 2019-05-23

**Authors:** Raheel Ahmad, Mehwish Changeez, Asim Tameez Ud Din, Anum Iftikhar, Hafiz Bilal Ahmad, Ahmed Mujtaba, Jahangir S Khan, Mustafa N Malik

**Affiliations:** 1 Surgery, Holy Family Hospital, Rawalpindi, PAK; 2 Internal Medicine, Rawalpindi Medical University, Rawalpindi, PAK; 3 Surgery, Fazaia Medical College, Islamabad, PAK; 4 Internal Medicine, The University of Arizona, Tucson, USA

**Keywords:** pain score, dexamethasone, thyroidectomy, post-operative nausea and vomiting, ponv

## Abstract

Introduction

Post-operative nausea, vomiting (PONV) and pain are the most frequently encountered complains after thyroid surgery. Steroids effectively reduce pain, nausea, and inflammation, therefore prophylactic administration of steroids improve these outcomes. The aim of our study was to compare the prophylactic administration of dexamethasone with placebo in terms of PONV and pain.

Patients and methods

We conducted a double-blinded randomized controlled trial including 100 patients who underwent thyroid surgery from January 2017 to December 2017 in Surgical Unit-I of the Holy Family hospital, Pakistan. The outcome in terms of post-operative pain, nausea and vomiting were measured.

Results

The mean age of the patients was 39.62 ± 12.73 years in group A, while in group B it was 39.06 ± 13.25 years. Out of the 100 patients included in our trial, 52 (52%) patients were males and 48 (48%) patients were females. The mean value of pain in group A patients was 1.60 ± 1.26, while in group B it was 3.60 ± 1.94. A statistically significant difference was found between the study groups with regard to the pain score of the patients i.e. *p*-value = 0.001. The PONV was found in 28 patients from group A and 19 patients from group B and no significant improvement was seen (*p*-value = 0.071).

Conclusion

A single dose of prophylactic dexamethasone significantly reduces the mean pain score in patients undergoing thyroidectomy; however, insignificant relation was noted in terms of PONV condition.

## Introduction

Thyroidectomy is one of the most prevalent endocrine surgeries done worldwide [1]. In the year 2007, 33,300 ambulatory and 39000 inpatient thyroidectomies were performed in the community hospitals of 28 states of the United States. In developing countries like Pakistan, thyroid diseases are also quite common especially in the northern areas, with females more commonly involved as compared to males (75.8% vs. 24.2%) [2]. Pain is one of the most common complains of patients of all type of surgeries and also an unavoidable complication of surgery [3]. Pain intensity after thyroidectomy can vary from mild to severe and can cause poor outcome in terms of post-operative surgical care. Another common adverse effect after surgery is postoperative nausea and vomiting (PONV). The incidence of PONV in general surgery is reported to be 20% to 30% which can rise up to 70% to 80 % if no prophylactic antiemetic therapy is given. These complaints not only reduce patient comfort but can also lead to serious postsurgical complications which can have detrimental effects on outcomes [4].

Dexamethasone, a glucocorticoid, has been described as extremely effective in reducing post-operative pain [5]. Not many studies have been published seeking the effects of a single-dose application of steroid before surgery in reducing pain and vomiting [6-7]. The opposing effect of steroids on inflammatory reactions may contribute to this effect [6]. The use of dexamethasone is found to be safe preoperatively for thyroid surgery. Feroci et al. showed that preoperative single dose of dexamethasone in patients who have undergone thyroidectomy reported significantly less pain (*p* = 0.008) and the need for analgesic medicines was less in the dexamethasone group (*p* = 0.048). Moreover, mean postoperative nausea and need for the use of the antiemetic drug was significantly decreased in the dexamethasone group as compared to control group (*p* = 0.0001) [7].

Pain and PONV after thyroid surgery are of the major concern not only for the patients but also for the surgeons. To date, only a few studies in the literature have been conducted to show the effect of dexamethasone on these postoperative complications after thyroid surgery. The aim of our study is to determine the efficacy of dexamethasone in preventing post thyroidectomy pain and PONV. 

## Materials and methods

Study design, setting, and duration

We conducted a double-blinded randomized controlled trial including 100 patients who underwent thyroid surgery from January 2017 to December 2017 in Surgical Unit-I of the Holy Family hospital, Pakistan.

Operational definitions

Pain Visual Analog Scale

Horizontal scale marked from zero to 100 after eight hours of surgery was used as a measure of VAS in pain [8].

Nausea and Vomiting

Nausea and vomiting were assessed after eight hours of surgery using a four-point scale (Table 1) [9].

**Table 1:** Four-point scale for nausea and vomiting assessment

Scale	Assessment
0	No nausea
1	Mild nausea defined as nausea requiring a single administration of an antiemetic drug
2	Severe nausea defined as nausea requiring the repeated administration of antiemetic drugs
3	Nausea leading to vomiting.

Inclusion and exclusion criteria

Our inclusion criteria are as follows: A) Patients aged between 18 to 60 years. B) Both males and females. C) Patients who are undergoing thyroidectomy for benign disease. Our exclusion criteria are as follows: A) Patients who had previous thyroid surgery, or neck surgery, chronic pain or necessity for sternotomy. B) Patients with a history of gastroesophageal reflux and acid peptic disease. C) Pre-operative vocal cord pathology.

Sampling technique

Non-probability consecutive sampling was done and all patients satisfying the inclusion criteria were included in our study. In Group A (study group), 8 mg/2 ml dexamethasone was injected intravenously thirty minutes before the induction of anesthesia, whereas in Group B (control group), two milliliters (ml) normal saline (0.9%) was given intravenously 30 minutes before the induction of anesthesia. To minimize the bias in our study, the patients and investigators were blinded to the allocation of patients to study group and to the types of injections administered. Surgery was standardized in each case by the same team of surgeons and the prophylactic antibiotics and preoperative and postoperative analgesia was also kept the standard in all patients. The completion of skin closure was considered as time point zero. Visual analog pain score was administered to assess the pain at eight hours post-surgery. Nausea and vomiting were also assessed at eight hours after surgery.

Data analysis

For statistical analyses, data were analyzed in a statistical package for social sciences (SPSS) 19. Categorical variables like gender were depicted as frequencies and percentages. Continuous variables such as age, visual analog pain score (VAS; zero-100) and PONV score (zero-3) was explained as mean and standard deviation. Independent sample t-test was used to compare the mean VAS and PONV scores of both study groups at five percent level of significance. A *p*-value of < 0.05 was considered statistically significant. A post-stratification independent sample *t*-test was applied. A *p*-value of ≤ 0.05 was taken as statistically significant.

## Results

In this study, a total of 100 patients were included. The mean age of the patients was 39.62 ± 12.73 years, while in group B, it was 39.06 ± 13.25 years (Table 2).

**Table 2:** Comparison of study groups according to the age of patients

	Group A (Dexamethasone)	Group B (Placebo)
Total Number of Patients	50	50
Age (Years); (Mean)	39.62	39.06
Age (Years); Standard Deviation (SD)	12.73	13.25

**Figure 1:**
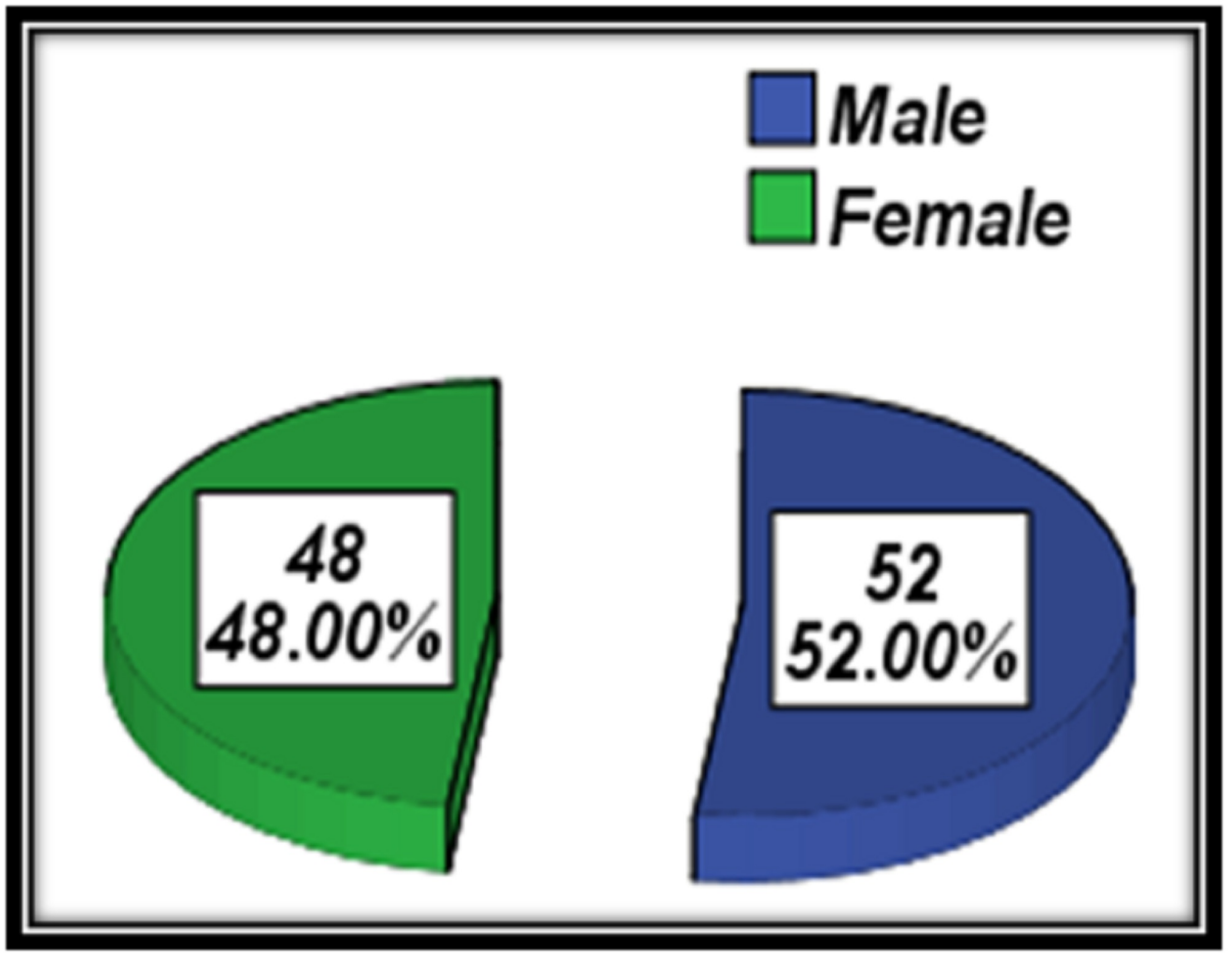
Distribution according to the gender of patients

Fifty-two percent of patients were males, while forty-eight percent of patients were females. Male to female ratio of the patients is shown in Figure 1.

Out of 52 male patients, 26 were from group A and 26 were from group B. Similarly, out of 48 female patients, 24 were from group A and 24 were from group B (Table 3).

**Table 3:** Comparison of study groups according to the gender of patients

Gender	Group A (Dexamethasone)	Group B (Placebo)	Total
Male	26	26	52
Female	24	24	48
Total	50	50	100

The study results showed that the mean value of pain in group A patients were 1.60 ± 1.26, while in group B, it was 3.60 ± 1.94. A statistically significant difference was found between the study groups in regards to the pain score of the patients (*p* = 0.001, Table 4).

**Table 4:** Pain score in the study groups A and B

	Group A (Dexamethasone)	Group B (Placebo)
Total number of patients	50	50
Pain score (Mean)	1.60	3.60
Pain score (SD)	1.26	1.94
t-test	6.12	
p-value	0.001	

**Figure 2:**
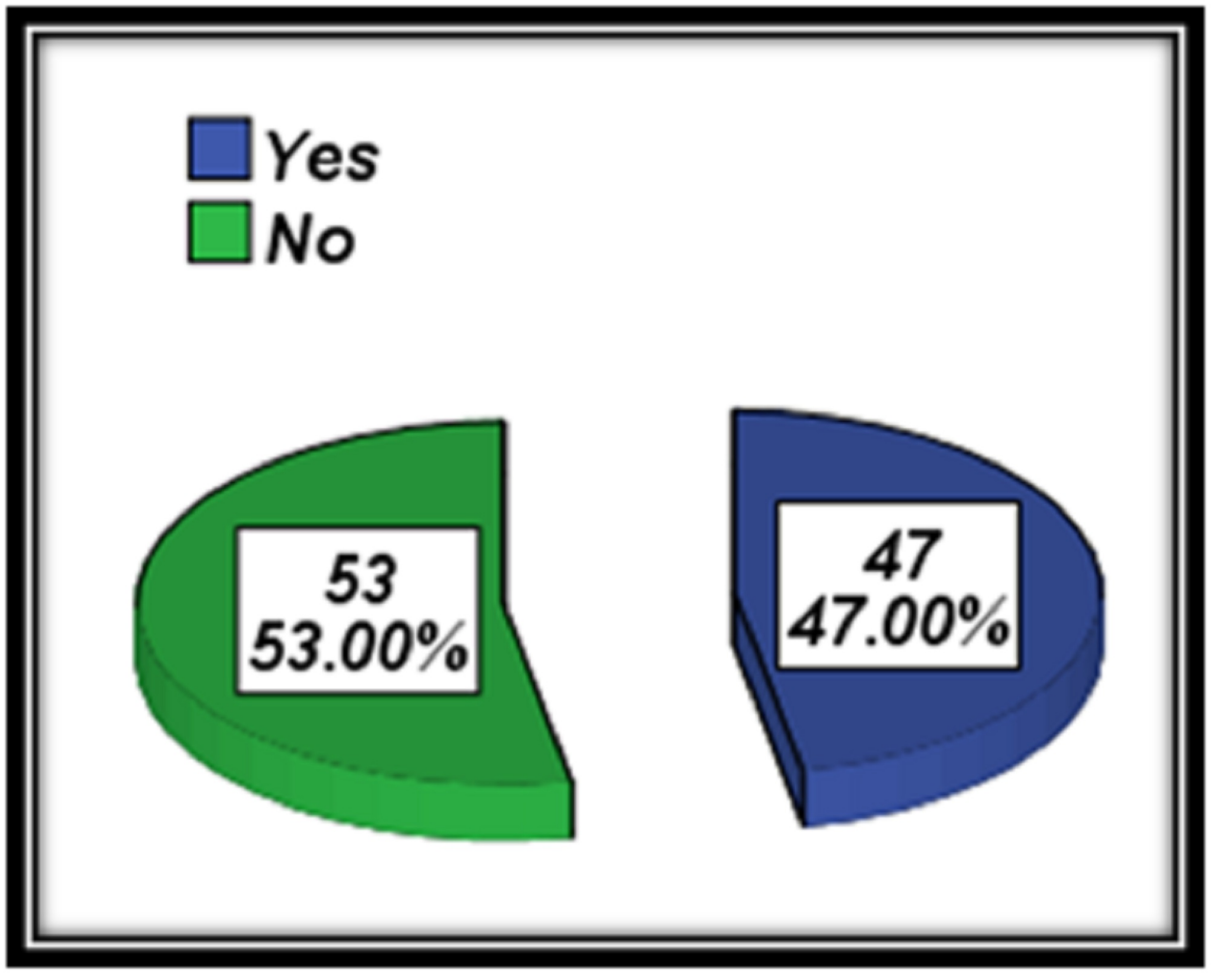
Frequency of PONV PONV, postoperative nausea and vomiting

Postoperative nausea and vomiting (PONV) were noted in 47 (47%) patients while it was not noted in 53 (53%) patients (Figure 2 & Table 5).

**Table 5:** PONV in the study groups A and B PONV, postoperative nausea and vomiting

PONV	Group A (Dexamethasone)	Group B (Placebo)	Total
Yes	28	19	47
No	22	31	53
Total	50	50	100
Chi value	3.25		
p-value	0.071 NS		

A statistically significant difference was found between the study groups in regard to the pain score of the patients stratified by age i.e. *p*-value = 0.001 & 0.003, respectively.

## Discussion

PONV and pain are the most common bothersome aftereffects of general surgery that have a negative impact on post-surgical outcomes. Although not life-threatening, these symptoms can be severe enough to delay early postoperative mobility and oral intake. Currently, there is ongoing research on various strategies that can counteract pain or at least mitigate it. Dexamethasone is a widely used corticosteroid that has been shown to influence patient and clinician-based outcome measures of recovery. It has been carefully assessed in the prevention of PONV in low/intermediate risk surgery and has shown to have a significantly great benefit [10].

In our study, the PONV condition was noted in 47 (47%) patients. Out of these, 28 patients were given dexamethasone while 19 patients were given placebo. The mean value of pain in dexamethasone group patients was 1.60 ± 1.26, while in placebo group patients, it was 3.60 ± 1.94. A statistically significant difference was found between the study groups with a pain score of the patients, i.e., *p*-value = 0.001. Chen CC et al. in his study demonstrated that preoperative dexamethasone decreased the incidence of PONV (relative risk [RR] 0.38; 95% confidence interval [CI] 0.30-0.49) and analgesic drug needs in patients undergoing thyroidectomy (RR 0.61; 95% CI [0.41-0.90]). Prophylactic use of corticosteroids for patients undergoing thyroidectomy is safe and should be considered routinely before such procedures [11].

Feroci et al. in his study demonstrated that preoperative single dose of dexamethasone in patients who have undergone thyroidectomy reported significantly lesser pain (*p* = 0.008) and the need for analgesic drugs was lower in the steroid group (*p* = 0.048). Moreover, mean PONV after eight hours in the dexamethasone group was significantly lower as compared to the control group (*p *= 0.0001) [7].

Allen TK et al. demonstrated that dexamethasone reduced the incidence of postoperative nausea (RR 0.57; 95% CI [0.45, 0.72]), vomiting (RR 0.56; 95% CI [0.43, 0.72]) and the use of additional antiemetic therapy (RR 0.47; 95% CI [0.36, 0.61]). There was no finding of dose responsiveness with respect to its antiemetic effect. Furthermore, dexamethasone also reduced 24-hour pain scores and the use of rescue analgesic drugs [12]. Worni M et al. in his study revealed similar outcomes that preoperative one shot of steroid reduced nausea, vomiting, and pain, and improved postoperative voice function within the first 48 hours after thyroid resection [13].

Another study by Li B et al. showed that preoperative corticosteroid treatment reduced the PONV, but not pain severity and analgesic requirement in patients. A statistically and clinically significant difference in the incidence and severity of postoperative nausea and vomiting was found in favor of dexamethasone, *p* < 0.00001 and *p* = 0.04, respectively. Surprisingly, there was no statistically significant difference in the reduction of pain severity and analgesic consumption [14].

A meta-analysis documented a significant reduction in the incidence of PONV, the need for additional anti-emetics (P < 0.00001), post-operative pain scores (P = 0.002), and the need for supplementary analgesics (*P* = 0.0008) in patients receiving dexamethasone compared to placebo. Moreover, a higher dose of dexamethasone (8-10 mg) had a significantly more effect in minimizing the incidence of PONV than the lower dose (1.25-5 mg) [15].

Trials like West Midlands Research Committee and the famous DREAMS Trial Collaborators concluded that the addition of a single dose of 8 mg of intravenous dexamethasone at the time anesthesia induction significantly reduces both PONV at 24 hours and the need for additional antiemetic medication for the period of up to 72 hours [10].

## Conclusions

Prophylactic dexamethasone significantly reduces the mean pain score in patients undergoing thyroidectomy. However, no statistically significant relation was noted in terms of postoperative nausea and vomiting. Data on preoperative use of dexamethasone in these patients is emerging and larger randomized prospective clinical trials are needed.
